# Allogeneic BMT in ATL: whom to transplant and which modality to use

**DOI:** 10.1186/1742-4690-11-S1-O32

**Published:** 2014-01-07

**Authors:** Mari Kannagi

**Affiliations:** 1 Tokyo Medical and Dental University, Japan

## 

Allogeneic hematopoietic stem cell transplantation (HSCT) has been used for treatment of ATL in Japan since 90’s, mainly for the patients with acute or lymphoma types of ATL after chemotherapies. The 3-year overall survival of HSCT in ATL is 31-39%, and many of the survivals live without disease for a long time. However, the treatment-related mortality (TRM) of HSCT is also high. Graft-versus-host (GVH) response is likely involved in both anti-tumor effects and TRM. Immunological study indicates the potential involvement of Tax-specific CTL in long-lasting anti-tumor surveillance after HSCT as well, suggesting that a tumor-vaccine to activate these CTL might partly reproduce the long-lasting effects of HSCT and could be an alternative choice of therapy following other initial therapies. The clinical trial of immunotherapy with Tax-peptide pulsed dendritic cells, currently undergoing in collaboration of National Kyushu Cancer Center, Kyushu University, and Tokyo Medical and Dental University, will find the answer.

